# Population structure of avian malaria parasites

**DOI:** 10.1002/ece3.5356

**Published:** 2019-06-17

**Authors:** Meghann B. Humphries, Matthew T. Stacy, Robert E. Ricklefs

**Affiliations:** ^1^ Department of Biology University of Missouri–Saint Louis St. Louis Missouri

**Keywords:** AMA1, avian malaria, *Haemoproteus*, Haemosporida, host–parasite genetic relationship, introgression, *Plasmodium*, spatial population structure

## Abstract

The geographic distribution of genetic diversity in malaria parasite populations (Apicomplexa: Haemosporida) presumably influences local patterns of virulence and the evolution of host‐resistance, but little is known about population genetic structure in these parasites. We assess the distribution of genetic diversity in the partial Domain I of apical membrane antigen 1 *(AMA1)* in three mtDNA‐defined lineages of avian *Plasmodium* to determine spatial population structure and host–parasite genetic relationships. We find that one parasite lineage is genetically differentiated in association with a single host genus and among some locations, but not with respect to other hosts. Two other parasite lineages are undifferentiated with respect to host species but exhibit geographic differentiation that is inconsistent with shared geographic barriers or with isolation‐by‐distance. Additional differentiation within two other lineages is unassociated with host species or location; in one case, we tentatively interpret this differentiation as the result of mitochondrial introgression from one of the lineages into a second lineage. More sampling of nuclear genetic diversity within populations of avian *Plasmodium* is needed to rule out coinfection as a possible confounding factor. If coinfections are not responsible for these findings, further assessment is needed to determine the frequency of mitonuclear discordance and its implications for defining parasite lineages based on mitochondrial genetic variation.

**OPEN RESEARCH BADGES:**

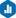

This article has earned an Open Data Badge for making publicly available the digitally‐shareable data necessary to reproduce the reported results. The data is available at Genbank https://www.ncbi.nlm.nih.gov/genbank/, accession numbers MK965548‐MK965653 and MK929797‐MK930264.

## 1 INTRODUCTION

The ubiquity of avian malaria in the global avifauna makes it an accessible pathogen to investigate how the geographic distribution of parasite genetic diversity relates to patterns of virulence, the evolution of host‐resistance, and the population structure and demography of hosts. Although more than 40 morphospecies of avian *Plasmodium* have been described (Valkiūnas, 2004) and hundreds of genetic lineages have been identified utilizing variation at the partial cytochrome *b* gene (Bensch, Hellgren, & Pérez‐Tris, 2009), little is known of the genetic diversity within avian malaria parasite populations, or even concerning the boundaries of these populations. Whether individuals of a particular parasite lineage vary genetically relative to their hosts or to geographic location is poorly understood, but assessments of lineage distributions through time reveal dynamic patterns that differ among both location and host taxa (Fallon, Bermingham, & Ricklefs, 2003; Fallon, Ricklefs, Swanson, & Bermingham, 2003). Previous work in the West Indies has revealed disjunctions in the geographic distributions of parasite lineages (Ricklefs, Soares, Ellis, & Latta, 2016) and dynamic parasite community assembly processes, including differentiation of geographically separated parasite assemblages in as little as 2,500 years (Soares, Latta, & Ricklefs, 2017). However, information about the genetic diversity and structure of parasite populations, including effective population sizes and intra‐lineage variation related to host species or geographic location, has not been reported. This lack of information reflects, in part, the difficulty of obtaining suitable genetic markers.

Genetic variation and population structures of two *Plasmodium* parasites infecting humans, *P*. *vivax* (*Pv*) and *P. falciparum* (*Pf*), have been characterized in detail, particularly at immunogenic loci. Among these loci is the apical membrane antigen 1 (*AMA1*), which plays a critical role in forming the junction between *Plasmodium* merozoites and host red blood cells (reviewed in Bai et al., 2005). *AMA1* is also thought to interact directly with host immune systems; analyses of *PfAMA1* have located one T‐cell epitope and a second B/T‐cell epitope in Domain I (Escalante et al., 2001), as well as several erythrocyte binding sites (Zakeri, Sadeghi, Abouie Mehrizi, & Dinparast Djadid, 2013). Analyses of variation in the three‐dimensional structure of the protein suggest that the acquisition of several highly variable loops in Domain I is related to evasion of the host immune response (Collins, Withers‐Martinez, Hackett, & Blackman, 2009). These direct interactions support Domain I of *AMA1* as a marker related to host‐specific immune pressures. Accordingly, the rapid accumulation of mutations as a result of diversifying selection provides information about fine‐scale population structure. Assessments of *PvAMA1* and *PfAMA1* reveal distinct demographic patterns: *PvAMA1* typically exhibits a differentiated structure consistent with an endemic pathogen (Neafsey et al., 2012; Taylor et al., 2013), while *PfAMA1* most often exhibits an epidemic population structure with reduced diversity and little geographic differentiation (Arnott et al., 2014; Mueller, Kaiok, Reeder, & Cortés, 2002; Ord, Tami, & Sutherland, 2008), typical of frequent clonal outbreaks. *Plasmodium vivax* and *P. falciparum* exhibit these demographic differences despite infecting the same hosts in many of the same locations and being transmitted by the same vectors. Therefore, populations of avian malaria parasites might be expected to exhibit substantial variation in the degree of population structure within lineages as a result of the more complex relationships among hosts, vectors, and locations.

To develop a better understanding of the influence of host species and geographic location on parasite genetic diversity, we assess the distribution of genetic diversity in the partial Domain 1 of *AMA1* of three mitochondrial lineages of avian *Plasmodium* commonly infecting birds in the West Indies and eastern North America. We assess genetic diversity and phylogenetic relationships within mitochondrial lineages and test whether parasite populations exhibit genetic structure related to hosts, location, or geographic distance.

## 2 MATERIALS AND METHODS

### 2.1 DNA extraction and sequencing

**Figure 1:**
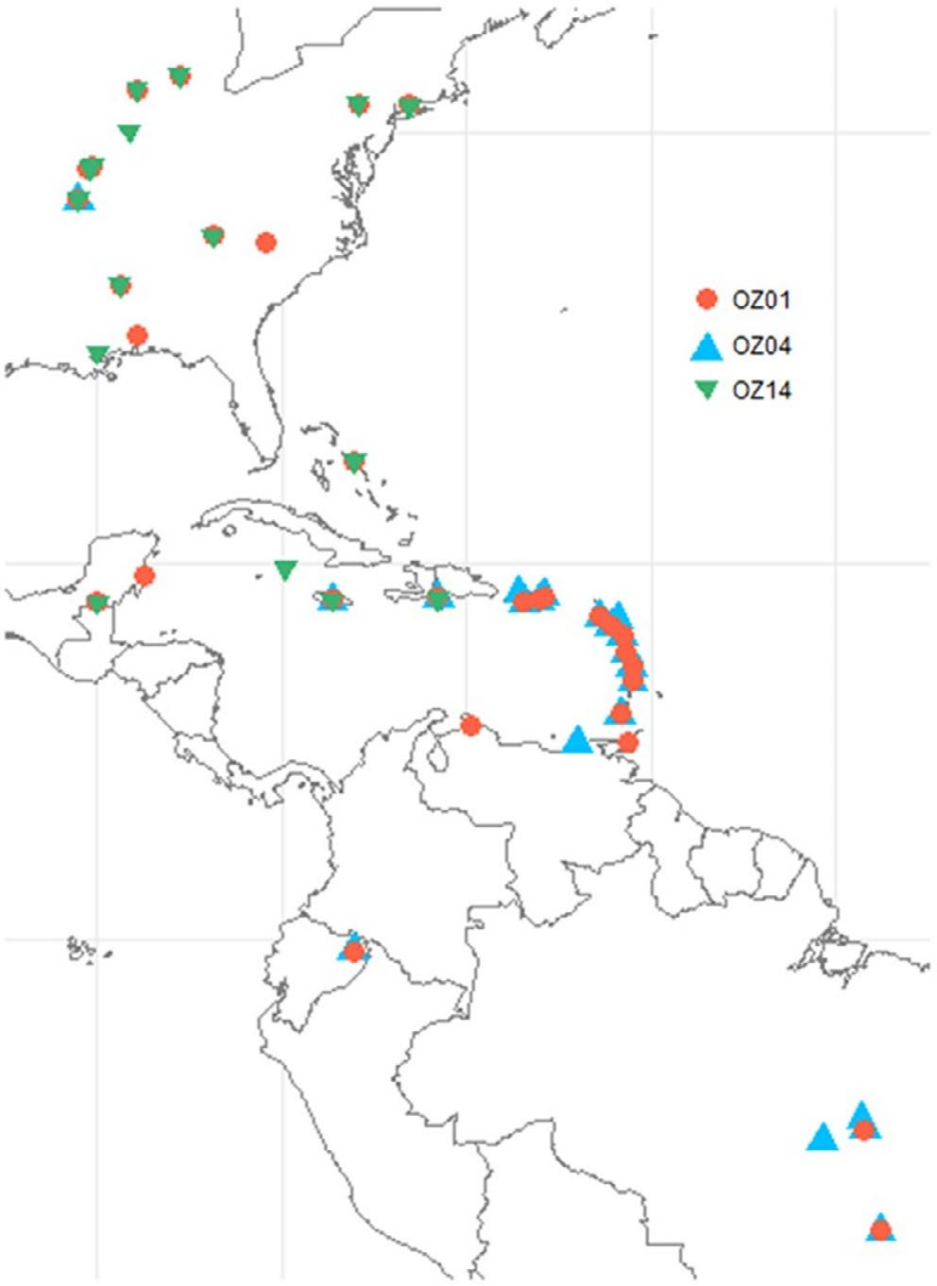
Overall detections of lineages OZ01 (blue triangles), OZ04 (red diamonds), and OZ14 (green circles). OZ01 is broadly distributed in the Neotropics and Nearctic; OZ04 is primarily restricted to the West Indies with limited detection in North and South America; OZ14 is distributed in the eastern United States and locally in the Greater Antilles. All three lineages are known to co‐occur on Jamaica, Hispaniola, and in the Ozarks region of Missouri

Samples for this study were obtained over several years from diverse localities in the Americas (see Figure 1). Birds were captured with mist‐nets and ca. 10 μl of blood was collected by sub‐brachial venipuncture (field techniques described in Latta & Ricklefs, 2010). DNA was extracted from blood using the isopropanol precipitation technique described in Svensson and Ricklefs (2009), and all samples were screened for avian malaria using primers 343F and 496R (Fallon et al., 2003, 2003). Samples that screened positive were genotyped to identify the cytochrome *b* lineage of the infection (Bensch et al., 2009) using a variety of primers and PCR conditions described in Perkins and Schall (2002), Ricklefs et al. (2005), and Waldenström, Bensch, Hasselquist, and Östman (2004). For samples infected by *Plasmodium* lineages OZ01 (equivalent to PADOM11 in the MalAvi database; Bensch et al., 2009), OZ04 (MalAvi ICTCAY01), and OZ14 (MalAvi CARCAR11), approximately 400 bp (length varies by lineage) of the partial Domain 1 of *AMA1* were amplified using nested primers Pg_AMA1F1/Pg_AMA1R1 and Pg_AMA1F2/Pg_AMA1R2 and PCR protocols described in Lauron et al. (2014). Negative controls were included in each PCR reaction, and products were verified by visualization on 1% TBE agarose gels with ethidium bromide. PCR product was cleaned using the ExoSAP‐IT protocol (Bell, 2008) and sequenced by Eurofins Genomics (Louisville, KY). Contigs were aligned and edited in Mega6 (Tamura, Stecher, Peterson, Filipski, & Kumar, 2013), and chromatograms were checked by eye to confirm polymorphisms. Heterozygous positions were denoted with IUPAC ambiguous base codes, and haplotypes were reconstructing using PHASE in DNAsp v. 5 (Librado & Rozas, 2009).

### 2.2 Statistical analyses of variation

Summary statistics describing genetic variation within parasite populations, based either on geography or host species, were calculated using DNAsp v.5 (Librado & Rozas, 2009). These statistics include number of haplotypes (*h*), haplotype diversity (*Hd*), and nucleotide diversity (π). *Hd* is the probability that two haplotypes chosen at random differ (Nei, 1987), while π is the average number of nucleotide differences per site for two sequences chosen at random (Nei & Li, 1979). We calculated π for the entire sequence, and to examine patterns of nucleotide substitution within the *AMA1* gene, we additionally calculated π using a sliding window of 30 bp and step size of 9 bp (Lauron et al., 2014). Synonymous and nonsynonymous substitutions, and minimum number of recombination events (*Rm*, using the four‐gamete test after Hudson & Kaplan, 1985), were also calculated in DNAsp v.5. Pairwise *F*_ST_ values were calculated in Arlequin v. 3.5 (Excoffier & Lischer, 2010) for a reduced dataset containing only sequences from locations or hosts with 10 or more samples.

### 2.3 Phylogeographic analysis

A median‐joining haplotype network was generated using Popart (Leigh & Bryant, 2015) to compare and visualize relationships among haplotypes at each sample location and within each host species or family. Pairwise *F*_ST_ values were calculated with confidence intervals based on 1000 permutations (Arlequin v.3.5, Excoffier & Lischer, 2010) for all locations with more than 10 sequences and for the two clusters identified in the haplotype networks of lineages OZ01 and OZ14. A Mantel test comparing pairwise *F*_ST_ and geographic distance was employed to determine whether the population structure detected among some locations in lineage OZ01 is consistent with isolation‐by‐distance. The Mantel test was implemented in the R package “ade4” version 1.7‐11 with 9,999 permutations.

## 3 RESULTS

### 3.1 Statistical analyses of variation

**Figure 2:**
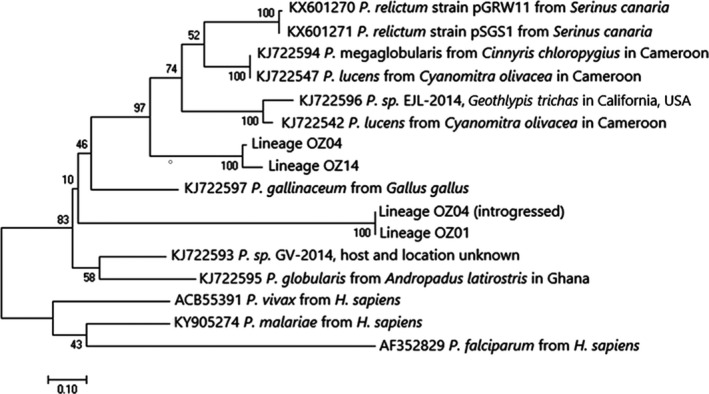
Phylogenetic relationships among the lineages assessed here and other *Plasmodium* species, based on a maximum likelihood phylogeny of the amino acid sequences produced in Mega6. In accordance with Mega6 model selection, the amino acid substitution model LG + G (Le & Gascuel, 2008) was implemented with five gamma categories and 100 bootstrap replicates. Included in the phylogeny are representatives of nine avian and three mammalian malaria species for which *AMA1* sequences were available (Genbank accessions KJ722597, KX601270, KX601271, KJ722596, KJ722593, KJ722594, KJ722595, KJ722542, KJ722547, KY905274, ACB55391, and AF352829)

We sequenced 389–407 base pairs (depending on lineage, Table 1) of the partial Domain 1 of *AMA1* corresponding to amino acid positions 169–295/300 of *P. falciparum* (3D7 isolate, Genbank accession U33274.1). A maximum likelihood phylogeny of *AMA1* amino acid sequences (Figure 2), including representatives of four avian and three mammalian *Plasmodium* species in addition to the three lineages assessed here, is concordant with the mitochondrial *CYTB* gene tree reported in Ricklefs et al. (2014, figure S2), recovering OZ04 and OZ14 as being closely related to each other relative to OZ01.

**Table 1:** *AMA1* sample information and summary statistics for lineages OZ01, OZ04, and OZ14. Major OZ04 includes only the major cluster, and Minor OZ04 includes only the apparently introgressed samples

	OZ01	OZ04	Major OZ04	Minor OZ04	OZ14
Number of sequences	232	170	108	62	134
Number of haplotypes	18	22	10	12	10
Haplotype diversity	0.607	0.717	0.362	0.804	0.430
Number of host species	31	16	12	12	23
Number of locations	18	11	9	8	12

**Figure 3:**
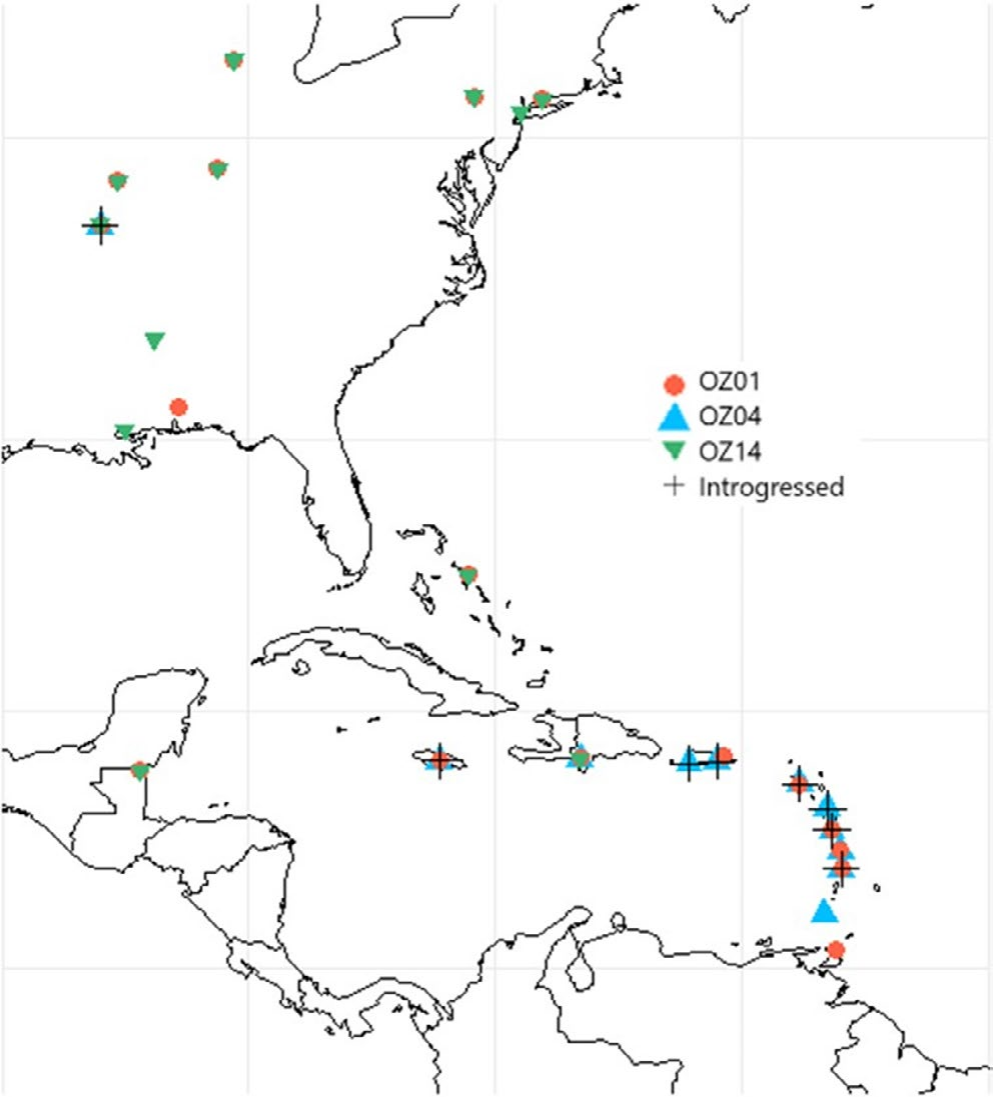
*AMA1* sampling localities depicting lineage co‐occurrences and locations of suspected introgression (crosses). Introgressed parasites were detected at seven localities where OZ01 and OZ04 are known to co‐occur and one locality where OZ01 has not been detected (Refugio Boquerón, PR)

Analysis of lineage OZ01 included 232 nucleotide sequences containing 18 unique haplotypes from 31 host species and 18 locations distributed in the eastern United States and the West Indies (Table 1, Figure 3). Analysis of the geographically more restricted lineage OZ04 included 170 nucleotide sequences containing 22 unique haplotypes from 16 host species and 11 locations distributed in the Lesser Antilles and in the Ozarks region of Missouri (Table 1, Figure 3). Lineage OZ14 comprised 134 nucleotide sequences representing 10 unique haplotypes from 23 host species and 12 locations distributed in the eastern United States and the West Indies (Table 1, Figure 3). Estimates of overall nucleotide diversity (*π*) were 0.004, 0.173, and 0.007 for OZ01, OZ04, and OZ14, respectively, and estimates of *Hd* were 0.607, 0.717, and 0.430 for the same sequences (Table 2). Lineage OZ01 exhibited the highest heterozygosity with 13 individuals possessing at least one heterozygous position; lineages OZ04 and OZ14 contained 3 and 7 such individuals, respectively (Table 3).

**Table 2:** Nucleotide diversity (π), percentage of synonymous variation (S%), and recombination estimates (Rm) for avian and human *Plasmodium* lineages

	OZ01	OZ04	Major OZ04	Minor OZ04	OZ14	*P. lucens*	*P. vivax*	*P. falciparum*
π	0.004	0.173	0.006	0.004	0.007	0.043/0.003^a^	~0.016^b^	0.01^e^
							0.0174^c^	0.0098^f^
S %	25	12.7	21.6	21.5	20.7	22^a^	24^d^	–
Rm	3	9	1	1	1	0^a^	9^d^	3^g^

Note

Lineage OZ04 estimates of π are for all sequences, including suspected cases of introgression, major OZ04 includes only the major cluster, and minor OZ04 includes only the apparently introgressed samples. Locations of samples for congeneric comparisons:

a Africa (for all sequences above, for a reduced dataset excluding seven sequences of uncertain taxonomic identity below, from Lauron et al., 2014),

b Venezuela (Grynberg et al., 2008),

c Brazil (Figtree et al., 2000),

d Sri Lanka (Gunasekera et al., 2007),

e Iran (Abouie Mehrizi, Sepehri, Karimi, Djadid, & Zakeri, 2013),

f 10 locations distributed globally (Escalante et al., 2001),

g Venezuela (Ord et al., 2008).

**Table 3:** *AMA1* genotype heterozygosity for lineages OZ01, OZ04, and OZ14, and for the suspected introgressed group

	OZ01	OZ04	OZ14	Introgressed group
Heterozygous Individuals	13	3	7	13
No. of ind. With 1 heterozygous position	12	1	3	13
No. of ind. With 2 heterozygous positions	1	1	4	0
No. of ind. With 3 heterozygous positions	0	1	0	0

**Figure 4:**
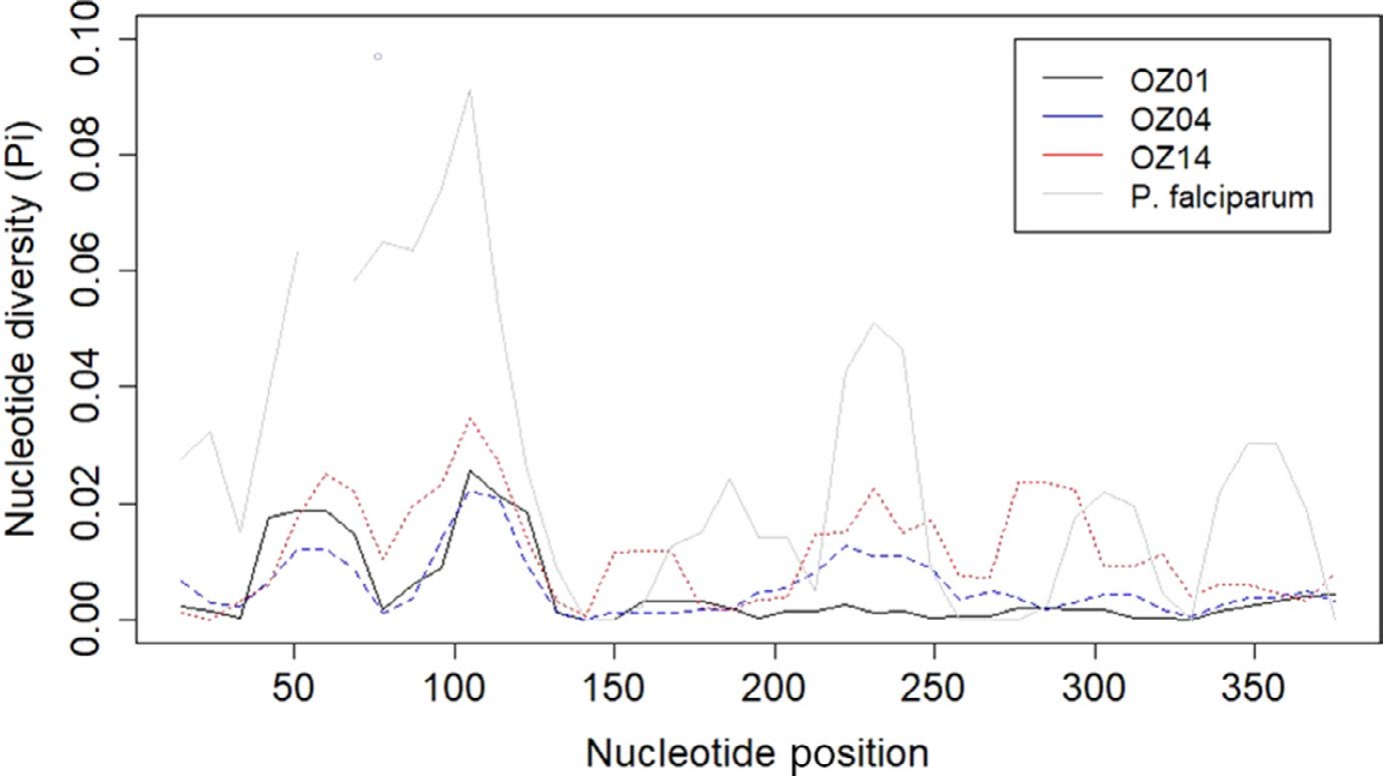
Sliding window analysis of nucleotide diversity for OZ01, OZ04, OZ14, and *Plasmodium falciparum* from Escalante et al. (2001). Window, 30 bp; step size, 9 bp

Sliding window analysis of π revealed congruence in the distribution of diversity among lineages, particularly within the range of nucleotides from positions 1–130 (Figure 4). We detected the highest polymorphism at nucleotide positions (nt) 94–130. This region aligns with *PfAMA1* amino acid residues 259–271 (Escalante et al., 2001), which constitute a hypervariable region encompassing a T‐cell epitope. Lineage OZ14 exhibits three additional peaks in π; one is shared with OZ04 (nt 204–249 in both lineages) and corresponds to a B/T‐cell epitope in *PfAMA1* at amino acid residues 279–288, a third is at *OZ14AMA1* nt 150–170, and the final peak is at *OZ14AMA1* nt 276–303. The latter two are not known to have immunogenic functions. These lineages share an amino acid insertion detected in other avian malaria parasites at *PfAMA1* amino acid residue 188 (Lauron et al., 2014), and lineage OZ01 and a subset of OZ04 exhibit a second amino acid insertion at the same position. Most of the nucleotides that encode a hydrophobic trough hypothesized to contain a critical structural component of the molecule are conserved; amino acid residues at *PfAMA1* tyrosine Y251, valine V169, and leucine L357 (Bai et al., 2005) are conserved in all samples, and *PfAMA1* phenylalanine F183 is conserved in all samples of lineages OZ01 and OZ04, although we detected three haplotypes with a serine at this position in lineage OZ14.

### 3.2 Phylogeographic analysis

**Figure 5:**
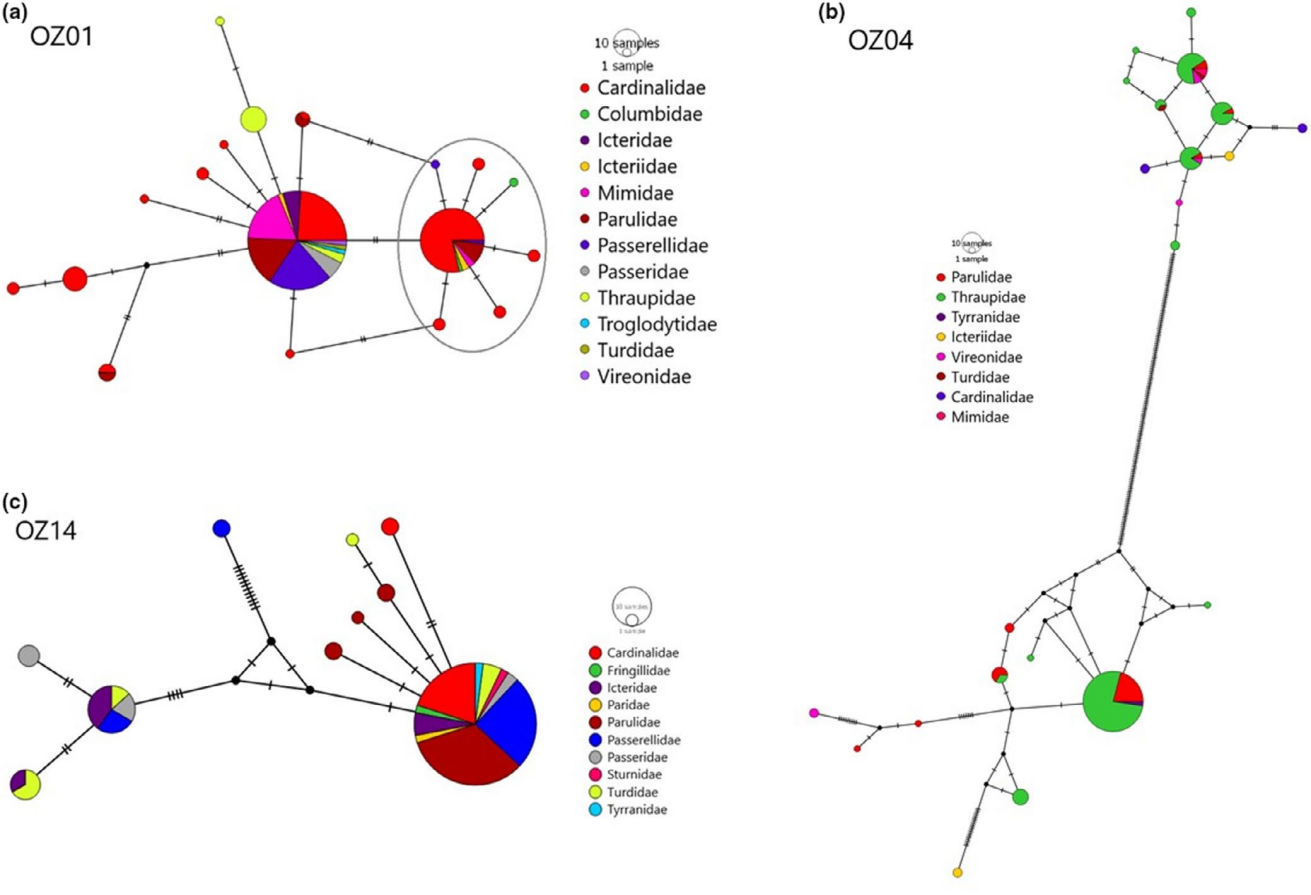
Median‐joining haplotype networks of *AMA1* variation for mitochondrial lineages OZ01 (A), OZ04 (B), and OZ14 (C). Circle size indicates the number of individuals with a given haplotype, color indicates host family/superfamily, and black hash marks indicate mutations. The gray oval in panel A encloses the group primarily infecting *Passerina*

Haplotype networks (Figure 5) reveal that lineages OZ04 and OZ14 each contain a lineage‐specific common and widespread *AMA1* haplotype, and that OZ01 contains two common haplotypes. However, in OZ01 these haplotypes are shared with individuals belonging to the mitochondrial OZ04 lineage (visualized in Figure 5A). One of the OZ01 haplotypes corresponds to a group of parasites infecting many hosts in many locations, and a second corresponds to a group infecting predominantly individuals of the avian genus *Passerina* (buntings): 42 of 68 infections in this group were detected in *P. cyanea,* and 6 of 68 were detected in *P. ciris*, representing 70.5% of the infections in this group and 87.5% of all *Passerina* OZ01 infections.

**Figure 6:**
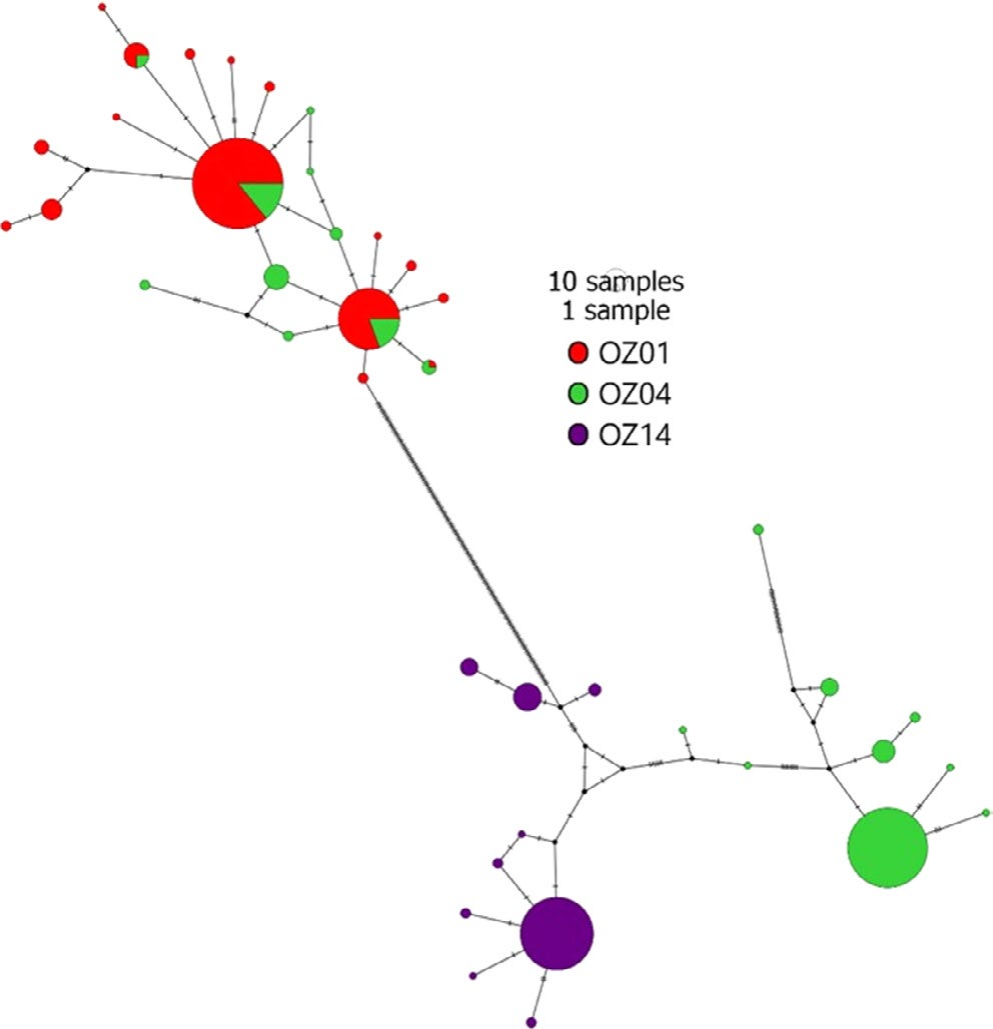
Median‐joining haplotype networks of *AMA1* depicting combined lineages OZ01, OZ04, and OZ14. Circle size indicates the number of individuals with a given haplotype, color indicates mitochondrial lineage designation, and black hash marks indicate mutations

Much of the diversity of OZ04 can be attributed to the presence of a single deep division within the lineage (visualized in Figure 5b). One subdivision of OZ04 consists primarily of a single common, widespread *AMA1* haplotype; the other subdivision includes haplotypes identical or nearly identical to several haplotypes detected in lineage OZ01 (Figure 6). Inspection of 21 (of 31 total) of the *CYTB* sequences of discordant samples showed no nucleotide variation from the reference sequence for lineage OZ04, indicating that the samples were correctly assigned to the lineage and supporting the absence of this divergence in the mitochondrial genome, though SNP variation for the remaining 10 samples was unavailable.

The *Passerina*‐dominant group within lineage OZ01 is significantly differentiated from the other group (*F*_ST_ = 0.74, *p* < 0.001), but pairwise *F*_ST_ values among the parasites of other well‐sampled hosts were not significant in any of the lineages assessed. The smaller group of *AMA1* haplotypes within OZ14 (visualized in Figure 5C) is significantly differentiated from the larger group in this lineage (*F*_ST_ = 0.88, *p* < 0.001) and does contain a preponderance of hosts in the family Icteridae (30% of this group, comprising 57% of the icterid infections sampled) but is broadly geographically distributed and exhibits no statistically significant differentiation in association with any one host species.

Support for differentiation among at least some locations comes from pairwise comparisons in each lineage. Lineage OZ01 exhibits a complex pattern of differentiation and gene flow (Table 4) while both OZ04 and OZ14 are differentiated with respect to only a single location: OZ04 samples from Saint Lucia are differentiated from those from Dominica, Guadeloupe, and Jamaica, but no other pairwise comparisons among these locations are significant; OZ14 samples from Chicago are differentiated with respect to samples from locations in Saint Louis, the Missouri Ozarks, Michigan, and Pennsylvania, but exhibit no other significant pairwise differences among these locations. Further investigation into the complex pattern of OZ01 revealed no shared geographic boundary among pairwise haplotype comparisons and no signal of isolation‐by‐distance (Mantel statistic *r*: −0.188, significance 0.69).

**Table 4:** Pairwise *F*_ST_ among locations for lineage OZ01 (only locations with 20 or more detections are included in this analysis), *N = s*ample, *p*‐value < 0.05 indicated by + and *p*‐value > 0.05 indicated by ‐ in upper diagonal. Note: negative *F*_ST_ value reported as 0

	*N*	Michigan	Illinois	Indiana	Penn.	Mexico	Ozarks	St. Louis
Michigan	20		−	+	−	+	+	+
Illinois	32	0		+	+	+	+	+
Indiana	34	0.484	0.5		+	+	+	+
Penn.	24	0.088	0.09	0.302		+	−	+
Mexico	26	0.253	0.277	0.119	0.121		+	−
Ozarks	34	0.297	0.303	0.1	0.082	0.067		−
St. Louis	22	0.289	0.315	0.083	0.137	0.019	0.048	

## 4 DISCUSSION

The avian malaria parasite lineages assessed here have been defined based on their similarity at the partial cytochrome *b* gene, but whether they represent good phylogenetic species has not been determined. Broad host ranges, propensity for host switching, and difficulty in linking morphological species and genetic lineages have all contributed to the problem of delimiting species in this group (Galen, Nunes, Sweet, & Perkins, 2018; Martinsen, Perkins, & Schall, 2008; Outlaw & Ricklefs, 2014). Moreover, a cut‐off based on percentage of divergence is not a useful way to define these species because it has been demonstrated through combined geographic range, host range, and corroboration with multiple loci that good phylogenetic species can exhibit low divergence at *CYTB* (Nilsson et al., 2016; Outlaw & Ricklefs, 2014). In the absence of morphological data, species delimitation is thought to be best addressed by comparing multiple gene trees (Bensch, Pérez‐Tris, Waldenström, & Hellgren, 2004; Galen et al., 2018; Outlaw & Ricklefs, 2014).

The *AMA1* gene tree produced here contradicts the mitochondrial lineage assignment in some cases. Specifically, all parasites from mitochondrial lineage OZ01 possess *AMA1* alleles similar to one another, but 31 parasites from mitochondrial lineage OZ04 also possess *AMA1* alleles identical or very similar to OZ01 alleles; the remaining parasites of lineage OZ04 possess *AMA1* alleles that are similar to one another and more than 60 mutations divergent from the OZ01 cluster (Figure 6). We interpret these relationships as supporting mitochondrial introgression from OZ04 to OZ01, but an alternative explanation of undetected coinfections cannot be ruled out with currently available data. Avian malaria coinfections are known to occur and are known to be underestimated by PCR as a result of preferential primer binding on variations of the template sequence (Bernotiene, Palinauskas, Iezhova, Murauskaite, & Valkiunas, 2016). Therefore, it is possible that the suspected introgressed parasites are instead OZ01/OZ04 coinfections in which our *CYTB* primers only amplified templates from OZ04 and our *AMA1* primers only amplified templates from OZ01.

**Figure 7:**
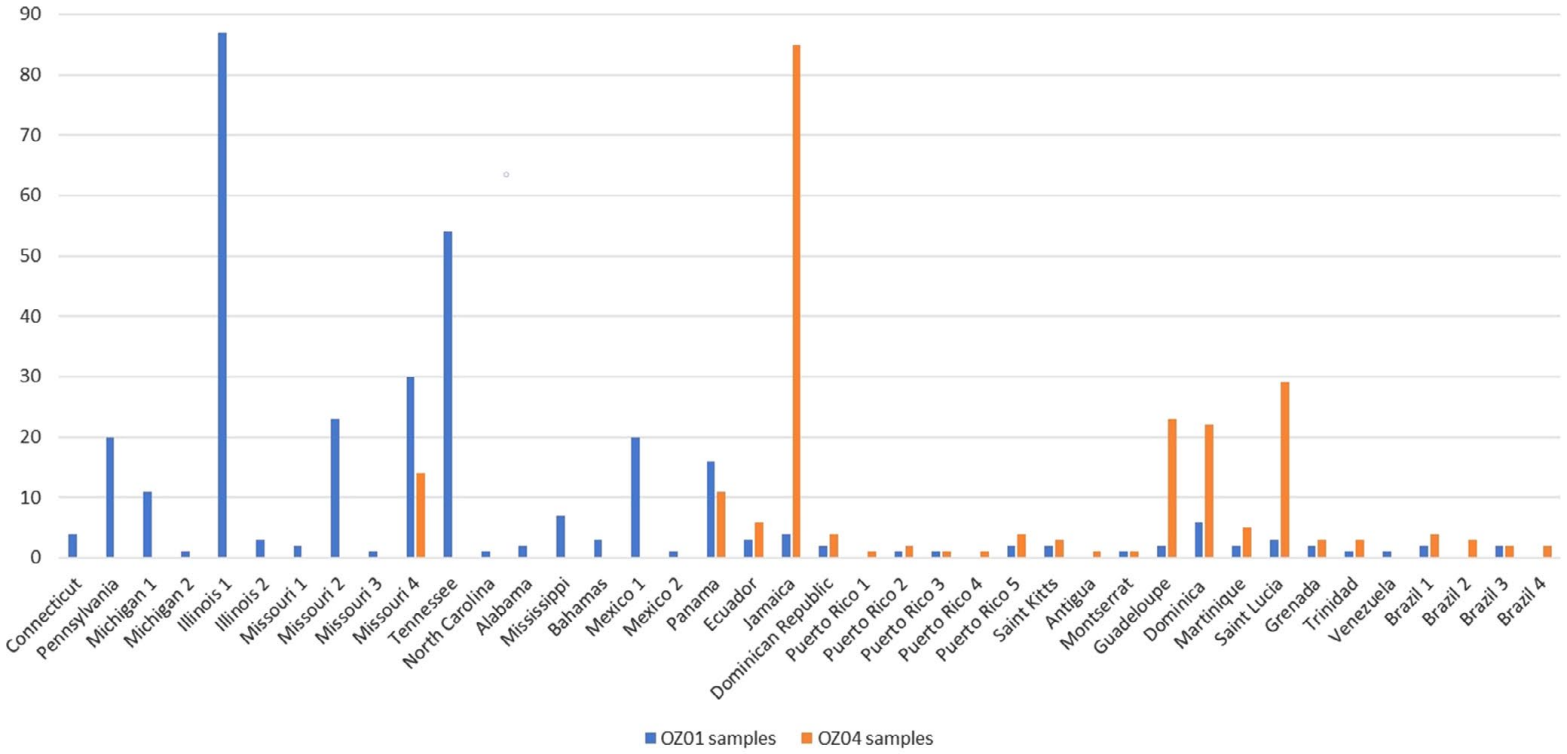
Number of detections by location for lineages OZ01 and OZ04

**Figure 8:**
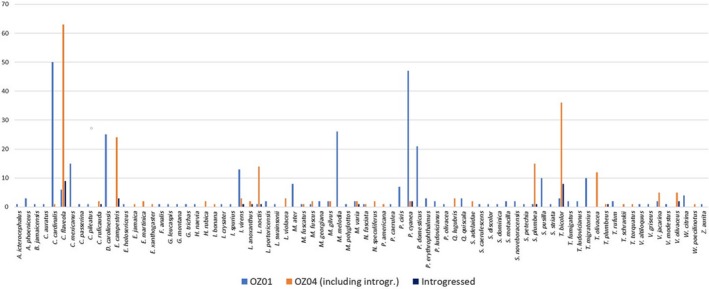
Number of detections by host species for lineages OZ01 and OZ04

**Figure 9:**
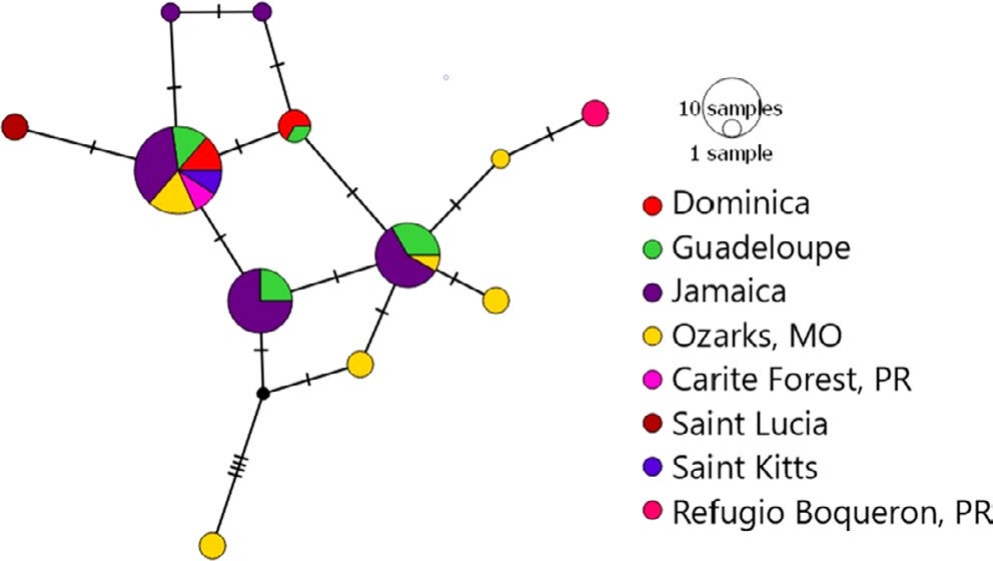
Median‐joining haplotype networks of *AMA1* for only introgressed samples. Circle size indicates the number of individuals with a given haplotype, color indicates location, and black hash marks indicate mutations

Individual *CYTB* SNP data were available for 21 (of 31) samples with suspected introgressed *AMA1* variants and revealed no difference from the reference sequence for lineage OZ04 (Genbank accession GQ395669) confirming appropriate lineage assignment and supporting our inference of introgression. We did not detect similar patterns between OZ14 and either of the other lineages, though they co‐occur in some of the same hosts and locations and might therefore be expected to exhibit similar rates of coinfection. Additionally, we recovered a suspected introgressed parasite in Refugio Boquerón, PR, where OZ01 has not been detected (it has been detected at very low abundances in other locations on Puerto Rico, see Figures 3 and 7), making it less likely to be a coinfection in this case. The lineages assessed here have not yet been assigned to morphospecies (Malavi database, Bensch et al., 2009), and a morphological assessment of lineages OZ01 and OZ04 following the protocols and keys described in Valkiūnas, Iezhova, Loiseau, and Sehgal (2009) and Valkiūnas and Iezhova (2018) revealed no discernible difference between lineages. As a result, coinfections could not be identified or ruled out at present by inspecting blood smears. Though we cannot definitively rule out coinfections in these cases, we proceed in the following with a tentative interpretation of mitochondrial introgression among lineages and encourage increased attention to this matter in future research. The introgressed OZ01 parasites detected here were most often recovered in hosts that are rarely parasitized by OZ01 but commonly parasitized by OZ04, namely *Coereba flaveola* and *Tiaris bicolor*. Thraupidae species host only 6% of detected OZ01 infections (7/116 detections, Figure 8) but make up 71% of the cases of evident introgression (22/31 detections, Figures 5b and 8), with *C. flaveola* and *T. bicolor* together accounting for 77% of these (8 and 6, respectively). These patterns suggest that mitochondrial introgression from OZ04 to OZ01 may have facilitated this host shift. Moreover, the higher relative abundance of introgressed OZ01 parasites on Jamaica, the co‐occurrence of OZ01 and OZ04 on the same island, and the relationship of the Jamaican haplotypes to the other introgressed haplotypes detected (Table 5, Figures 7 and 9) support Jamaica as the likely site of introgression. One possible hypothesis for this occurrence is that an individual mosquito vector on this island was infected by both OZ01 and OZ04 gametes (from either multiple blood meals or a single co‐infected host) which hybridized during sexual reproduction in the mosquito vector. The hybrid progeny then repeatedly backcrossed with OZ01 parasites to produce the mitonuclear discordance we detect in contemporary populations.

**Table 5:** Host and geographic location of introgressed parasites

	JAM	OZ	GU	DO	CF‐PR	RB‐PR	SK	SL	Sum
*Coereba flaveola*	7						1	1	9
*Tiaris bicolor*	2		3	1	1	1			8
*Euneornis campestris*	3								3
*Passerina cyanea*		2							2
*Vireo olivaceus*		2							2
*Cinclocerthia ruficauda*			1						1
*Setophaga plumbea*			1						1
*Icteria virens*		1							1
*Loxipasser anoxanthus*	1								1
*Loxigilla noctis*			1						1
*Mniotilta varia*		1							1
*Turdus plumbeus*				1					1
Sum	13	6	6	2	1	1	1	1	31

Location abbreviations are as follows: JAM, Jamaica; OZ, Ozarks region of Missouri; GU, Guadeloupe; DO, Dominica; CF‐PR, Carite Forest, Puerto Rico; RB‐PR, Refugio Boqueron, Puerto Rico; SK, Saint Kitts; SL, Saint Lucia.

The introgressed parasites exhibit several geographic disjunctions that might be related to migratory movement of hosts. Specifically, one or more migrants might have carried the parasite from Jamaica to the North American Midwest (e.g., Missouri Ozarks), where it was transmitted to other hosts, which then carried it to Puerto Rico and the Lesser Antilles in following migrations, skipping the island of Hispaniola. Further dispersal within the Lesser Antilles may have been facilitated by movements of common endemic species, including *C. flaveola* and *T. bicolor*. Other intra‐lineage divisions, for example in lineage OZ14 and in *P. lucens* (Lauron et al. (2014), discussed below), may also represent mitonuclear discordance resulting from introgression, although further sampling will be required to substantiate this. If substantiated, these findings imply that defining lineages based on *CYTB* similarity might often be problematic.

**Figure 10:**
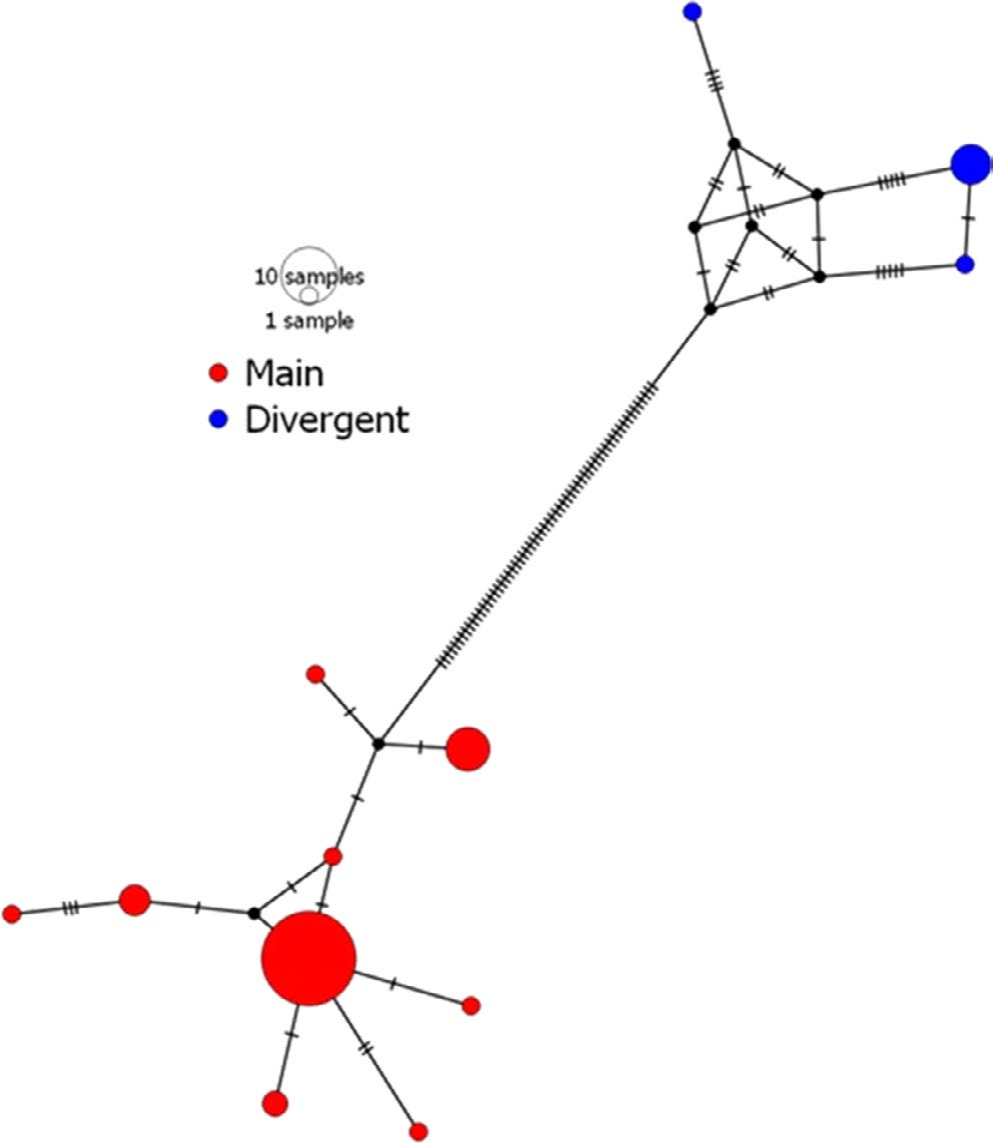
Median‐joining haplotype network of 51 *Plasmodium lucens AMA1* sequences depicting the main group (red) and the divergent group (blue). All samples were recovered from a population of olive sunbirds (*Cyanomitra olivacea*) in Cameroon (Lauron et al., 2014)

The comparatively high estimate of nucleotide diversity (π) for samples of *P. lucens* prompted our further investigation into the relationships among *AMA1* haplotypes within that parasite species. We constructed a median‐joining haplotype network for *P. lucens* (Figure 10) which revealed that seven of the 51 sequences (three of 12 haplotypes) cluster together more than 60 mutational steps from the other haplotypes. The pairwise nucleotide differences among haplotypes within the major cluster vary between one and three substitutions, suggesting uncertain taxonomic identity of the minor cluster. If the divergent sequences are removed from analysis, the estimate of *π* is 0.003 (reported in Table 2), consistent with findings for OZ01 and OZ14. The estimate for lineage OZ04 provided here is also exceedingly high, but expectedly so because it includes the highly divergent introgressed group. Removal of the divergent group in this case produced an estimate of *π* = 0.006 for lineage OZ04.

We found no support for isolation‐by‐distance in the widespread lineage OZ01, and lineages OZ04 and OZ14 are genetically undifferentiated with respect to host species. OZ04 does, however, exhibit overall reduced host breadth compared to OZ14, with the former infecting mainly West Indian tanagers such as bananaquits (*Coereba flaveola*), grassquits (*Tiaris bicolor*), and bullfinches (*Loxigilla* spp.), while the latter infects extremely diverse hosts. The lack of population differentiation among some locations in OZ01 and OZ14 is likely related to host dispersal as these lineages infect primarily migratory species. In contrast, we detected OZ04 most commonly in resident species. The presence of genetic differentiation among locations within lineages might be promoted by geographic isolation related to migratory paths of hosts or vector dispersal, but we lack information to assess these possibilities at present.

### 4.1 Congeneric comparisons

*Plasmodium vivax* and *P. falciparum* represent contrasting demographic patterns and impacts on hosts, and these parasites may provide an informative context in which to consider the findings presented here. In general, *P. vivax* causes less mortality and is more likely to persist at low densities and with a lower transmission rate than *P. falciparum* (Neafsey et al., 2012). *Plasmodium vivax* has more diverse populations, exhibits dormancy and relapse, and dominates the interepidemic periods when sympatric with *P. falciparum* (Arnott et al., 2014; Neafsey et al., 2012). *Plasmodium falciparum* exhibits a contrasting demographic pattern typified by dramatic clonal outbreaks (Mueller et al., 2002; Razakandrainibe et al., 2005) and more severe host illness. The avian parasites with the three *AMA1* lineages described here exhibit population patterns inconsistent with either the epidemic pathogen structure of *P. falciparum* or the endemic pathogen structure of *P. vivax*. That is, our lineages present comparatively low diversity at *AMA1*, as in *P. falciparum,* but nonetheless exhibit local differentiation and recombination as in *P. vivax*. Little is known of other avian malaria parasite populations, but analysis of a taxonomically conservative subset of *P. lucens AMA1* from a population of olive sunbirds (*Cyanomitra olivacea*) in Cameroon (Lauron et al., 2014) is consistent with our finding that genetic diversity in the avian parasites is lower than both *P. vivax* (Figtree et al., 2000; Grynberg, Fontes, Hughes, & Braga, 2008) and *P. falciparum* (Escalante et al., 2001).

## 5 CONCLUSIONS

Analyses at more loci and with wider taxon sampling will be necessary to uncover the complexities of these relationships, but findings here provide a glimpse into the host distribution, spatial distribution, and diversity of avian malaria *AMA1* in natural host communities. The complex spatial patterns and differentiation in relation to host genus described here suggest several possible influences on population structure, including host immune pressure, host dispersal, and migratory movements, as well as vector dispersal and host feeding preferences. Moreover, the mitonuclear discordance detected here warrants further investigation to assess the role of coinfections and to determine the frequency of such introgression events and implications for defining parasite lineages based on mitochondrial genetic variation.

## CONFLICT OF INTEREST

All authors declare no conflict of interest.

## AUTHOR CONTRIBUTIONS

RER and MBH designed the research; RER collected samples; MBH and MTS performed laboratory and statistical analyses; MBH wrote the manuscript with contributions and revisions by all authors. All authors approved the final manuscript.

## Data Availability

All sequences deposited to Genbank, accession numbers MK965548‐MK965653 and MK929797‐MK930264.
